# Cancer care cannot wait: oncology system resilience in the UAE during regional conflict in the gulf

**DOI:** 10.3389/fonc.2026.1833847

**Published:** 2026-04-30

**Authors:** Humaid O. Al-Shamsi, Deborah Mukherji, Saeed Rafii

**Affiliations:** 1 Burjeel Cancer Institute, Burjeel Medical City, Abu Dhabi, United Arab Emirates; 2 Department of Medical Oncology , Dana-Farber Cancer Institute and Harvard Medical School, Boston, MA, United States; 3 College of Medicine, Ras Al Khaimah Medical and Health Sciences University, Ras Al Khaimah, United Arab Emirates; 4 College of Medicine, Gulf Medical University, Ajman, United Arab Emirates; 5 College of Medicine, University of Sharjah, Sharjah, United Arab Emirates; 6 Emirates Oncology Society, Emirates Medical Association, Dubai, United Arab Emirates; 7 Clemencue Medical Centre, Dubai , United Arab Emirates; 8 Mediclinic Hospital, Dubai, United Arab Emirates

**Keywords:** cancer care, conflict, crisis, healthcare system (HCSys), oncology

Cancer care follows biological timelines, not geopolitical ones. For patients with cancer, interruptions to chemotherapy, radiotherapy, or surgery—even if brief—can compromise curative potential and adversely affect survival. Yet, armed conflicts and geopolitical instability continue to disrupt healthcare systems worldwide, disproportionately affecting patients with cancer, who are among the most vulnerable during crises (1).

Evidence from multiple conflict zones illustrates the fragility of oncology services during war. In Syria, widespread destruction of healthcare facilities during the protracted conflict severely disrupted cancer diagnosis and treatment pathways (2). In Ukraine, ongoing hostilities have interrupted radiotherapy services, delayed chemotherapy delivery, and forced many oncology patients to seek treatment abroad (3). Similar patterns have been observed in other fragile settings including Sudan and Iraq where shortages of oncology medications, damaged infrastructure, and displacement of healthcare workers have undermined cancer care delivery (4). These disruptions translate into delayed diagnoses, interrupted treatment, and poorer survival outcomes. Protecting cancer services during acute and prolonged crisis has become an increasingly urgent challenge for global oncology systems (2, 5–7).

The global burden of cancer continues to rise. Nearly 20 million new cancer cases were diagnosed worldwide in 2022, and projections suggest that this number will increase substantially over the coming decades (8). Additionally in the UAE young onset cancer diagnosis is significantly higher that the western population. The UAE Cancer registry reported that 25.4% of all cancers are diagnosed below the age 40 and 49% below the age of 49 in its 2021 report (9). These highlight that maintaining uninterrupted oncology services during crises must be recognised as a central component of health system resilience (5–7).

In crisis settings, proactive and patient-centred strategies are essential to safeguard continuity of cancer care. Clear and timely communication with patients and caregivers regarding hospital operations, treatment schedules, and potential disruptions—aligned with national and local government directives—is critical to maintaining trust and adherence to therapy. Oncology centres should ensure that all patients have access to comprehensive, portable medical records and clearly documented treatment plans to facilitate continuity of care should relocation or cross-border transfer become necessary. Psychological support services must be integrated into crisis response frameworks to address the compounded stress of cancer diagnosis and security concerns for both patients and their caregivers. Equally important is the protection and support of healthcare workers, whose wellbeing, safety, and operational capacity are fundamental to sustaining oncology services during periods of instability. Embedding these measures within institutional and national preparedness plans can enhance resilience and mitigate the impact of future crises on cancer outcomes. These principles align closely with emerging global frameworks for oncology care in conflict settings as discussed during the first Global Summit on War and Cancer 2024 (7). A structured approach to maintaining cancer services during wartime has been proposed, emphasizing continuity of treatment pathways, protection of healthcare infrastructure, secure pharmaceutical supply chains, and regional coordination of care delivery (6).

Recent security tensions and geopolitical instability in the Gulf region provide a contemporary context to reflect on oncology service continuity. This article presents an early expert perspective based on clinical observation and system-level insights, rather than a formal outcomes analysis. On Feb 28^th^, 2026; regional instability escalated following missile and drone attacks targeting infrastructure across the Gulf countries, and the UAE being the most affected country with numbers of daily attacks in various infrastructures in the country. Although national defence systems intercepted most threats, the events nevertheless highlighted the vulnerability of civilian infrastructure, including healthcare systems and pharmaceutical supply chains.

Despite these security concerns, oncology services in the United Arab Emirates (UAE) continued operating without major disruption. Outpatient oncology clinics remain open, systemic therapy infusions proceed according to schedule, and radiotherapy treatments continue as reported by individual institutions. Multidisciplinary oncology teams were able to maintain routine clinical operations, allowing patients to continue receiving planned treatments.

Several structural factors appear to have contributed to this resilience. Sustained national investment in healthcare infrastructure has strengthened the capacity of the UAE health system. Over the past two decades, the country has developed advanced tertiary cancer centres equipped with modern radiotherapy technologies, molecular diagnostics, and multidisciplinary oncology programmes (9). These centres operate within coordinated national healthcare systems overseen by regulatory authorities including the Ministry of Health and Prevention, the Abu Dhabi Department of Health, and the Dubai Health Authority, enabling rapid coordination during emergencies (9).

National emergency preparedness frameworks have also been integrated into healthcare operations. Hospitals function under established crisis-management protocols designed to maintain essential services during emergencies. These frameworks involve coordination with civil defence authorities, emergency medical services, and national crisis-management structures. Digital health infrastructure—including integrated electronic health records —facilitates continuity of care by enabling rapid communication across healthcare institutions.

Pharmaceutical supply chain resilience proved equally important. Oncology medications depend on complex international manufacturing and distribution systems and are therefore particularly vulnerable during geopolitical instability. In many conflict settings, disruptions to supply chains lead to chemotherapy shortages and treatment delays. In the UAE, pharmaceutical supply is overseen by the Emirates Drug Establishment, which regulates drug importation, distribution, and strategic stockpiling. During the recent regional tensions, oncology drug availability remained stable, allowing treatment protocols to continue without any interruption.

Equally critical is the resilience of the healthcare workforce. Physicians, nurses, pharmacists, and allied health professionals form the operational backbone of healthcare delivery during crises. In many conflict zones, workforce displacement or safety concerns significantly reduce hospital capacity. In contrast, healthcare professionals across the UAE (with the majority being expats living and working in the country) maintain full clinical operations during the period of regional instability, ensuring uninterrupted chemotherapy, radiotherapy, and supportive care.

Public trust in the healthcare system also played an important role. In the time of conflicts patients may choose to avoid hospital visits due to safety concerns or uncertainty about service availability. However, patients in the UAE continue attending scheduled oncology appointments and treatment sessions. Stable attendance at infusion centres and radiotherapy departments reflect strong public confidence in the UAE’s healthcare system stability.

Another important observation is the capacity of UAE cancer centres to absorb patients returning from overseas treatment (10). Travel disruptions prompted some patients who had previously planned to seek cancer care abroad to stay in the UAE. Local oncology centres were able to integrate these patients rapidly into treatment pathways without delaying therapy. This capacity reflects the growing maturity of the UAE oncology ecosystem, which has increasingly evolved into a regional hub for advanced cancer services.

The principles underlying oncology system resilience depend on complex systems that integrate specialised infrastructure, workforce capacity, and reliable pharmaceutical supply chains. Strengthening these components should therefore be central to national health security strategies worldwide (11).

Although conflicts have historically devastated healthcare systems and disrupted cancer services across many regions of the world, the recent experience of the UAE demonstrates that well-prepared health systems can maintain continuity of complex oncology care even during periods of instability. These observations should be interpreted as preliminary and hypothesis-generating, highlighting the need for systematic data collection and prospective evaluation of oncology service continuity during crises, including treatment delays, outcomes, and patient experience. Strong infrastructure, coordinated governance, resilient pharmaceutical supply chains, committed healthcare professionals, and sustained patient trust together form the foundation of oncology system resilience. Cancer does not pause for war—and neither should cancer care.
